# Authors’ reply

**Published:** 2010-06

**Authors:** Richa Vaishya, J. Singh, Harbans Lal

**Affiliations:** Department of Biochemistry, Pt. BD Sharma Postgraduate Institute of Medical Science, Rohtak, Haryana, India; 1 Department of Pharmacology, Pt. BD Sharma Postgraduate Institute of Medical Science, Rohtak, Haryana, India

Sir,

We thank Dr. Mahajan for his comments[1] on our paper.[2] Our reply to the queries are as follows:

1. The study was planned to focus the beneficial effects of irbesartan in comparison to insulin and thus the relevance of the study remains clear.
2. Suggestion at 2 is well-taken for future studies.
3. We regret the typing error; please read group III for insulin and group IV for irbesartan.
4. It is a printing error. The original Table (1 and 2) submitted to the journal office are reproduced here.

**Table 1:** Effect of irbesartan (20 mg/kg/po) pretreatment on urinary protein excretion in STZ-induced diabetic rats (values are mean ± SEM; n = 10 in each group)

*Group*	*Urine protein (g/L)*				
	*0 week*	*4 weeks*	*8 weeks*	*12 weeks*	*16 weeks*
I Control	Nil	Nil	Nil	Nil	Nil
IISTZ	Nil	1.0 ± 0.07	1.6 ± 0.16	1.9 ± 0.16	2.1 ± 0.13
IIIInsulin + STZ	Nil	Nil	Nil	Nil	Nil
IVIrbesartan + STZ	Nil	0.5 ± 0.04‡	0.8 ± 0.06‡	0.9 ± 0.08‡	0.8 ± 0.09‡

‡ *P* <0.01 when compared with group II (Student’s t-test for unpaired data).

**Table 2:** Effect of irbesartan (20 mg/kg, po) pretreatment on creatinine clearance in STZ-induced diabetic rats (values are mean ± SEM; n = 10 in each group)

*Group*	*Creatinine clearance (ml/min)*				
	*0 week*	*4 weeks*	*8 weeks*	*12 weeks*	*16 weeks*
I Control	1.1 ± 0.18	1.1 ± 0.09	1.1 ± 0.04	1.1 ± 0.05	1.2 ± 0.06
IISTZ	0.9 ± 0.07	1.8 ± 0.16	0.9 ± 0.12	0.2 ± 0.11	0.1 ± 0.03
IIIInsulin + STZ	0.9 ± 0.08	0.9 ± 0.07‡	0.8 ± 0.06	0.8 ± 0.08‡	0.7 ± 0.03‡
IVIrbesartan + STZ	1.0 ± 0.03	0.7 ± 0.03‡	0.6 ± 0.18	0.5 ± 0.15	0.4 ± 0.02‡†

Statistically analyzed by Student’s *t*-test (unpaired)

‡ *P* <0.01 when compared with group II (Student’s *t*-test for unpaired data).
